# Detection and Analysis of Corrosion and Contact Resistance Faults of TiN and CrN Coatings on 410 Stainless Steel as Bipolar Plates in PEM Fuel Cells

**DOI:** 10.3390/s22030750

**Published:** 2022-01-19

**Authors:** Mohsen Forouzanmehr, Kazem Reza Kashyzadeh, Amirhossein Borjali, Anastas Ivanov, Mosayeb Jafarnode, Tat-Hean Gan, Bin Wang, Mahmoud Chizari

**Affiliations:** 1 Department of Mechanical Engineering, Shahid Bahonar University of Kerman, Kerman 7618868366, Iran; foruozanmehr@gmail.com; 2 Department of Transport, Academy of Engineering, Peoples’ Friendship University of Russia (RUDN University), 6 Miklukho-Maklaya Street, 117198 Moscow, Russia; 3 Department of Mechanical Engineering, Sharif University of Technology, Tehran 11155-1639, Iran; a.borjali15@student.sharif.ir; 4 Department of Mechanics, Todor Kableshkov University of Transport, 158 Geo Milev Street, 1574 Sofia, Bulgaria; aii2010@abv.bg; 5 School of Mechanical Engineering, Islamic Azad University, South Tehran Branch, Tehran 1584743311, Iran; m.jafarnoudeh@gmail.com; 6 TWI Ltd., Granta Park, Great Abington, Cambridge CB21 6AL, UK; 7 College of Engineering and Physical Sciences, Brunel University London, Uxbridge UB8 3PH, UK; bin.wang@brunel.ac.uk; 8 School of Physics, Engineering and Computer Sciences, University of Hertfordshire, Hatfield AL10 9AB, UK; m.chizari@herts.ac.uk

**Keywords:** fuel cells, coating, corrosion, Interfacial Contact Resistance

## Abstract

Bipolar Plates (BPPs) are the most crucial component of the Polymer Electrolyte Membrane (PEM) fuel cell system. To improve fuel cell stack performance and lifetime, corrosion resistance and Interfacial Contact Resistance (ICR) enhancement are two essential factors for metallic BPPs. One of the most effective methods to achieve this purpose is adding a thin solid film of conductive coating on the surfaces of these plates. In the present study, 410 Stainless Steel (SS) was selected as a metallic bipolar plate. The coating process was performed using titanium nitride and chromium nitride by the Cathodic Arc Evaporation (CAE) method. The main focus of this study was to select the best coating among CrN and TiN on the proposed alloy as a substrate of PEM fuel cells through the comparison technique with simultaneous consideration of corrosion resistance and ICR value. After verifying the TiN and CrN coating compound, the electrochemical assessment was conducted by the potentiodynamic polarization (PDP) and electrochemical impedance spectroscopy (EIS) tests. The results of PDP show that all coated samples have an increase in the polarization resistance ($R_{p}$) values (ranging from 410.2 to 690.6 $\Omega \cdot {\mathrm{cm}}^{2}$) compared to substrate 410 SS (230.1$\;\Omega \cdot {\mathrm{cm}}^{2}$). Corrosion rate values for bare 410 SS, CrN, and TiN coatings were measured as 0.096, 0.032, and 0.060 mpy, respectively. Facilities for X-ray Diffraction (XRD), Scanning Electron Microscope (FE-SEM, TeScan-Mira III model and made in the Czech Republic), and Energy Dispersive X-ray Spectroscopy (EDXS) were utilized to perform phase, corrosion behavior, and microstructure analysis. Furthermore, ICR tests were performed on both coated and uncoated specimens. However, the ICR of the coated samples increased slightly compared to uncoated samples. Finally, according to corrosion performance results and ICR values, it can be concluded that the CrN layer is a suitable choice for deposition on 410 SS with the aim of being used in a BPP fuel cell system.

## 1. Introduction

As a result of the rising cost of fossil fuels and the pressing need for environmental protection, special attention has been paid to fuel cells as viable alternative energy sources [1]. BPPs are one of the essential components in PEM fuel cells, responsible for separating cells, electrically connecting the cathode of one cell to the anode of another cell, feeding reactive gases through gas channels, and removing heat and byproducts from the fuel cell [2,3]. Moreover, bipolar plates should possess unique characteristics, including non-permeability of gas, high mechanical strength, thermal conductivity, high corrosion resistance, mechanical and chemical stability, low Interfacial Contact Resistance (ICR), high electrical conductivity, and low cost [4,5].

**Figure 1:**
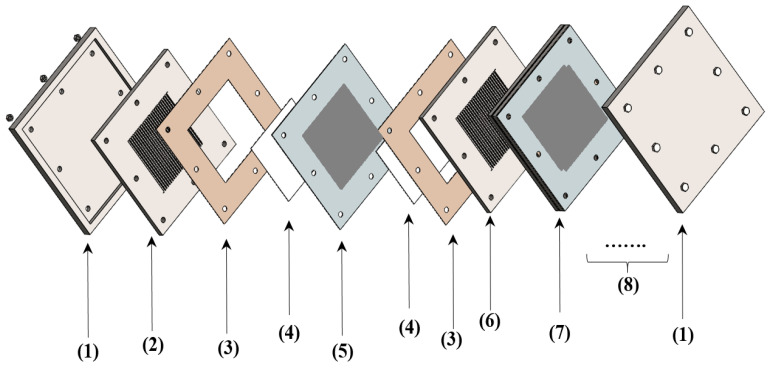
An exploded view of a PEM fuel cell stack: (1) clamping plates; (2) endplates; (3) gasket; (4) gas diffusion layer; (5) catalyst layer; (6) bipolar plate; and (7) stack.

Figure 1 depicts an exploded view of a PEM fuel cell stack. As can be seen, the primary structure of the fuel cell stack is connected to the BPPs. For this purpose, most commercial BPPs are fabricated of non-porous graphite due to its thermal and chemical stability in the PEM fuel cell components. However, the manufacturing costs of carbon-based bipolar plates, particularly non-graphite, are very high. Furthermore, graphite is brittle and has poor mechanical properties [6,7]. Therefore, researchers are interested in developing suitable substitutes for non-porous graphite to produce BPPs with the lowest total cost and increased electrical conductivity.

Metals are denser than composite or graphite, so an inherent reduction in their thickness is essential. Aluminum, nickel, copper, and titanium alloys are frequently used to manufacture BPPs [3,8,9]. Due to their superior mechanical properties and low gas permeation, stainless steels have been more commonly used in research [10]. However, the corrosion phenomenon and its associated ICR are the most challenging issues in metal bipolar plates [11,12]. In general, when SS plates are exposed to air at the anode, a thin inactive oxide layer (thickness~1–3 nm) forms, increasing the risk of PEM contamination and negatively affecting the catalyst layer activity [13]. At the cathode, due to the presence of an oxidizing environment, the corrosion rate can be inherently increased, resulting in performance losses or even premature failure of the entire stack [14,15]. Moreover, it causes an increase in the ICR between the bipolar plates and the gas diffusion layer [16]. According to the findings of other researchers and experimental studies, coating these plates is one of the most practical techniques for resolving this issue [17]. Extensive research has been undertaken to investigate the effects of different coatings such as CrMoN, PMAT/PYY, CrN/TiN, Ti/TiN, Ti, and TiN on corrosion resistance and the ICR value of substrate SS 316L, SS 904L, and SS 321L alloys as a bipolar plate in fuel cells [2,3,18,19,20]. Jin et al. investigated the effects of CrMoN coating deposited on SS 316L using closed field unbalanced magnetron sputter ion plating to improve ICR and corrosion resistance under PEMFCs. The results showed that the performance of CrMoN-coated SS 316L was 14 times greater than the bare substrate case, with a ICR of 11.2 $m\Omega .{\mathrm{cm}}^{2}$ [2]. Li et al. [21] and Cho et al. [22] demonstrated significant improvements in the ICR of 316L steel bipolar plates coated with TiN. Their findings indicated that bipolar plates with TiN coating, such as graphite, can rapidly remove water. Moreover, the TiN coating provides superior corrosion resistance and electrical conductivity compared to uncoated SS specimens. Forouzanmehr et al. analyzed the effects of TiN and CrN coatings of SS 410 bare samples on roughness and ICR [23]. They showed that the minimum surface roughness for CrN, TiN, and non-coated specimens is 182, 223, and 323 nm, respectively. Furthermore, they proposed CrN coating for bipolar plates in fuel cells based on the minimum value of ICR. However, due to the lack of study of the corrosion resistance effects on the proposed coatings and the expression of small differences in ICR, the selection process of the best coating among those proposed should be further investigated.

Yi. et al. deposited TiN and ZrN coatings on the surface through magnetron sputtering [12]. In this research, surface morphology, corrosion resistance, and ICR of coated samples were investigated. As a result of this research, it was determined that TiN coatings have a higher corrosion resistance than ZrN coatings using Electrochemical Impedance Spectroscopy (EIS) and polarization diagrams. Furthermore, contact angle experiments showed that both TiN and ZrN can increase the hydrophobicity of SS. Recently, to advance previous research, single-layer TiN/TiAlN and double-layer TiN/TiAlN coatings were deposited on 316L SS using the Chemical Vapor Deposition (CVD) technique [24]. In this study, the electrochemical corrosion behavior of both coated and uncoated specimens was evaluated in a solution comprising H_2_SO_4_ 0.5 M and 2 ppm of fluoride ion to simulate a proton-exchange membrane fuel cell environment. Based on the main findings of this study, the best protection performance against corrosion was found in TiN coating, TiN/TiAlN coating, uncoated specimen, and TiAlN coating, respectively. Brady et al. investigated the effect of chromium nitride coating on Ni-Cr and Fe-Cr alloys to optimize the efficiency of metal bipolar plates [25,26]. They used the nitration of commercial austenite alloys with high-chromium (30%–35%), Ni-Cr alloys, and ferritic alloys with 29% wt. of Cr as the bipolar plates. After 2700 h of operation in the fuel cell environment, under both anode and cathode conditions, heat nitration of SS with a high chromium content resulted in extremely low ICR values. They also reported that the amount of oxygen present during thermal nitration is critical for the structure of the formed nitride surface. Oxides form on the steel surface when oxygen is present during thermal nitration. However, they concluded that nitration of alloys with a high chromium content can reduce ICR and increase conductivity. Moreover, the corrosion resistance of CrN and Cr2N on the surface of these bipolar plates can meet the fuel cell requirements. Fu et al. optimized a chromium nitride coating on 316L steel for use as a bipolar plate in a proton exchange membrane fuel cell [27]. They used the pulsed bias arc ion plating method for the coating process. After conducting potentiostat and potentiodynamic tests at 70 °C in both anode and cathode environments, it was determined that the chromium nitride-coated samples exhibited significantly greater corrosion resistance than that of the uncoated samples. Moreover, the results indicated that the ICR of the coated samples is less than that of the uncoated targets. According to the literature, single-layer metal nitrides, which included titanium nitride (TiN), zirconium Nitride (ZrN), and chromium nitride (CrN) coating deposited by the PVD technique, demonstrated excellent electrical conductivity and corrosion resistance [3,28,29]. Despite the results of previous studies and the practical significance of various coatings on metal bases for BPPs, and given the growing importance identified by scientists in using fuel cells in daily life, it is necessary to understand the behavior of other materials, such as different substrates, in addition to the use of various coatings. Therefore, in this research, SS 410 was selected as the substrate, considering the most important factors for the fabrication of bipolar plates of PEM fuel cells, including high resistance and the substrate’s life. Moreover, by reviewing the literature, it can be inferred that TiN and CrN coatings are the most important coatings from an applied point of view in industry. Accordingly, comparing the performance of two common coatings, TiN and CrN, on the proposed alloy SS 410 in fuel cells is required. However, the selection of superior coatings on SS 410 has not been addressed via the simultaneous examination of corrosion resistance, ICR, and surface roughness. This research aimed to address this issue. For this purpose, the Physical Vapor Deposition (PVD) method in conjunction with arc cathode technology was used for the first time to coat 410 stainless steel bipolar plates in a simulated fuel cell environment. After specimen preparation, corrosion measurements were made using various techniques, including potentiodynamic polarization (PDP), and electrochemical impedance spectroscopy (EIS). Finally, the efficiency of the fuel cells with coated and uncoated 410 SS bipolar plates was determined, and their corrosion resistance and ICR were compared. To summarize, the latest developments and side-by-side comparisons of the literature on proposed coatings used in PEM are reported in Table 1.

## 2. Experimental Procedure

### 2.1. Material and BPP Sample

This study utilized 410 SS, which is martensitic stainless steel. It possesses greater mechanical strength and is less expensive than the austenitic stainless steels, which have been used in most research on metallic bipolar plates used in fuel cells [35]. Table 2 presents the chemical composition of the substrate metal. The Glow Discharge Optical Emission Spectrometer (GDOES) technique was used to obtain the results. Due to the soft nature of 410 series martensitic steel, it must be heat treated to increase strength, improve wear resistance, and reduce stresses caused by manufacturing processes. The raw material was quenched in an austenitic salt furnace at a temperature of 1050 °C and then in hot oil. Then, it was washed and dried when its temperature reached ambient temperature. Next, a tempering operation at 120 °C for 10 min was used to stress relieve the sheet. Then, the sheet reached ambient temperature and was thoroughly cleaned. In this case, the sheet hardness was determined to be 49 HRC using a Rockwell C hardness tester. Specimens were fabricated by wire cutting in dimensions of 10 × 10 mm^2^ and a thickness of 3 mm. Finally, the specimens were leveled using a magnetic grinding machine.

### 2.2. Coating Process

**Figure 2:**
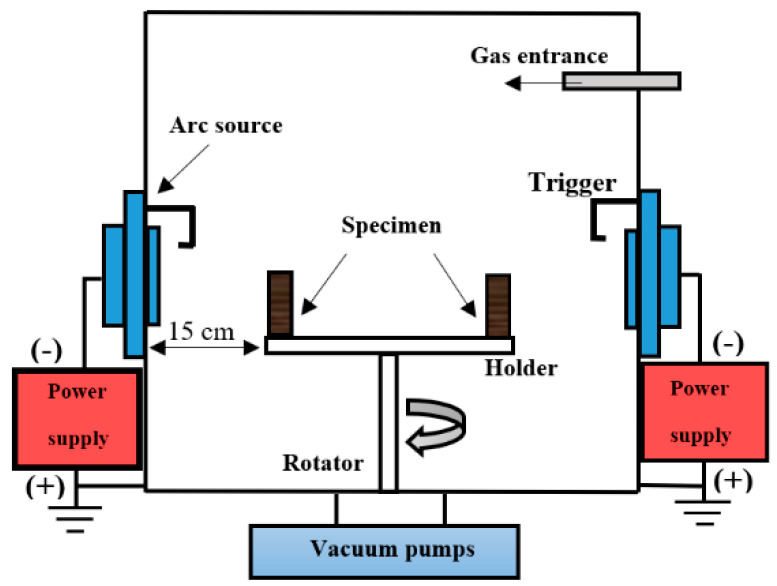
Schematic of the CAE deposition method.

The PVD method deposited the TiN and CrN coatings with Cathode Arc Evaporation (CAE) technology, which is a layering method using physical vapor deposition, in which the material is evaporated from the cathodic target with the help of an electric arc and the vaporized material can be made of ceramic, metal, and even composite films on the substrate [36], and one cathode arc head is employed. A schematic of the (CAE) deposition is shown in Figure 2. The target metal diameter, current, and voltage were 80 mm, 1000 A, and 40 V, respectively. Additionally, the distance from the arc head to the specimen was 30 cm. The initial vacuum before the coating was $10^{-6}$ mbar and the vacuum during coating was $10^{-3}$ mbar. Before coating, the surface of the specimens was subjected to argon ion bombing for 30 min using level 5 argon gas at a pressure of $10^{-2}$ mbar and −1000 V bias. Moreover, the bias voltage and specimen temperature during the coating were −80 V and 250 °C, respectively. Furthermore, the specimens rotated at a rate of 5 revolutions per minute around the central axis of the chamber and 30 revolutions per minute around their central axis.

### 2.3. Surface Characterization

In this part of the study, the crystallographic texture of the coating was analyzed. An X-ray diffractometer (JEOL JDX-8030) was used in the diffraction angle range of 2θ = 20° to 80° with CuKα radiation (λ = 1.54 Å) having a voltage and current of 30 kV and 20 mA, respectively. Moreover, SEM observations with a Philips EXCKW-30KW were used to examine the surface morphology and measure the coating thickness before and after the corrosion test. Moreover, EDX analysis was used to confirm the XRD results and the coating elements.

### 2.4. ICR Measurement Setup

**Figure 3:**
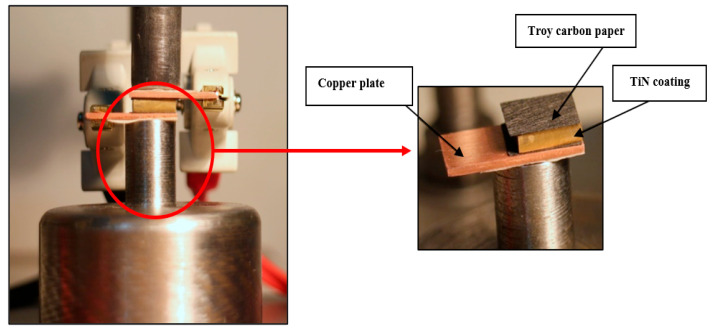
The experimental setup for the ICR measurement [23].

The technique proposed by Wang et al. [37] was considered for measuring the ICR between bipolar plates and the gas diffusion layer (GDL). For this purpose, a Lutron MO-2013 milliohm meter with an accuracy of 1 µΩ was used. Because the unit of contact resistance in polymer fuel cells is $m\Omega .{\mathrm{cm}}^{2}$, the use of a 1 ${\mathrm{cm}}^{2}$ sample is preferred to prevent dimensional conversion. A GDL (Toray TGP-H-060) with a thickness of 190 µm was used to simulate the fuel cell environment. The specimens were placed between two gas diffusion layers and then between two copper plates as electrodes. To prevent the formation of surface oxides, which causes errors in measuring the contact resistance, after each test, the copper surfaces were cleaned by #400 grit ultra-fine sandpaper with a polishing machine. Then, a current of 1 amp was passed through two copper plates, and the voltage difference between the two ends of the stack (copper plates) was measured. Moreover, the potential was measured to calculate the total resistance ($R_{T})$ of the GDL/bipolar plates assembly. As illustrated in Figure 3, a unique mechanical setup was used to apply force to this arrangement. The applied pressure during the ICR test was adjusted in the range of 35 to 150 N.cm^−2^, the total resistance of the setup was recorded by the milliohm meter, and each GDL layer was used only once. Moreover, to ensure the repeatability of the results, each test was repeated three times to increase the ICR test’s accuracy.

**Figure 4:**
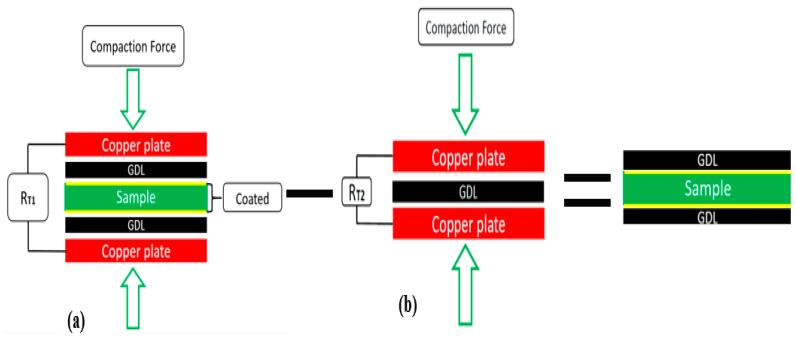
The arrangement schematic of the total resistances: (**a**) R_T1 and (**b**) R_T2.

To complete the above description, two different setups were used to measure the contact resistance of the bipolar plates coated with the GDL (Figure 4).

The total resistance ($R_{T1}$) of the schematic in Figure 4a was obtained by applying the compaction force and measuring $R_{T1}$ using a milliohm meter. The total resistance, which is given by Equation (1), can be calculated by the summation of the bulk resistance of two copper plates ($2R_{Cu})$, the GDL ($2R_{GDL}),$ the contact resistance between copper GDL and ($2R_{Cu-GDL}),$ the internal resistance (power source, resistance in the wire, $R_{i}$, etc.), and the contact resistance between BPPs and GDL ($2R_{BP-GDL})$.

(1) $$R_{T1}=2R_{Cu}+2R_{Cu-GDL}+2R_{GDL}+2R_{BP-GDL}+R_{BP}+R_{i}$$

To measure the value of $2R_{BP-GDL}$, in accordance with Figure 4b, the following equation was utilized.

(2) $$R_{T2}=2R_{Cu}+2R_{Cu-GDL}+2R_{GDL}+2R_{BP-GDL}+R_{BP}+R_{i}$$

Finally, by subtracting Equation (2) from Equation (1), the resistance $R_{BP-GDL}$ can be determined, as presented in Equation (3). In Wang’s method, the two terms of $R_{GDL}$ and $R_{BP}$ are neglected due to their small values.

(3) $$R_{BP-GDL}=0.5\left(R_{T1}-R_{T2}-R_{BP}-R_{GDL}\right)≅0.5\left(R_{T1}-R_{T2}\right)$$

### 2.5. Corrosion Resistance Measurement

PDP and EIS tests were used to determine the corrosion protection properties of the coated specimens. A Potentiostat/Galvanostat EG&G (273A model) was used for this purpose. All experiments were performed at the open circuit potential under atmospheric conditions at 25 °C using a three-electrode system, consisting of a calomel reference electrode, a platinum sheet as the auxiliary electrode, and coated specimens as the working electrode. Electrochemical tests were carried out in Ringer’s solution considering the conditions of free air and room temperature (25 °C). The chemical composition of this solution, which is widely used in biological applications for surgical instruments [38], is given in Table 3.

Additionally, the potential in the PDP test was performed from −0.8 VSCE to +1.5 VSCE with a scanning rate of 1 mV/s. The required data were recorded using Powersuite software. The EG&G was coupled to a Schlumberger (Si1250) FRA to measure the impedance [39]. The frequency range was between 65 kHz and 10 MHz during the EIS test, and the turbulence voltage was set to 10 mV. Before measurement, 30 min was allowed to ensure the results were stable. For both PDP and EIS tests, the engaged area of the working electrode was 0.785 cm^2^. Finally, the impedance data were analyzed using the Zview2 software [40].

## 3. Results and Discussions

### 3.1. XRD Analysis

**Figure 5:**
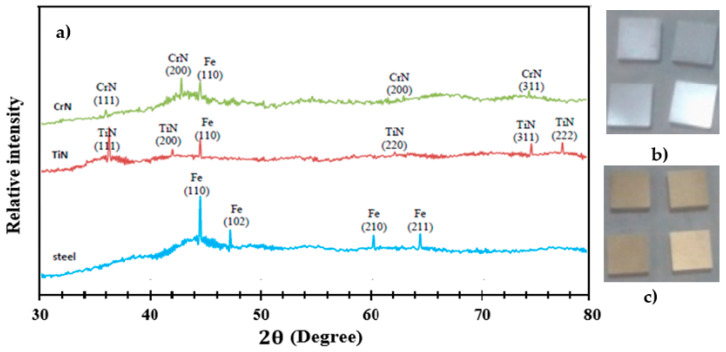
(**a**) XRD analysis results for coated and uncoated specimens, (**b**) TiN-coated samples, and (**c**) CrN-coated samples.

The results of the XRD test for different specimens are depicted in Figure 5a. In the diffraction pattern of coated specimens, the peak at the (110) plane is related to the steel substrate at a 42° angle. This is due to the greater penetration depth of the X-ray compared to the coating thickness, resulting in the diffraction of the steel substrate. In the diffraction pattern of the CrN-coated specimen, the peaks at the (111), (200), and (311) planes were observed, consistent with the standard diffraction pattern and reference pattern (2494-076-01) for CrN. The (111) plane has a high intensity, indicating that the coating’s preferred growth direction is in the (111) direction. The coating’s flat silver color (Figure 5b) and analysis of the XRD results compared to reference patterns confirm the presence of the CrN phase on the SS substrate. The coating demonstrates the cubic structure and polycrystalline nature of CrN. The peaks associated with the (111), (200), (220), and (311) planes are visible in the diffraction pattern of the TiN-coated specimen, which is consistent with the standard diffraction pattern and reference pattern (0642-006-00) of TiN. The (111) plane has a high intensity, indicating that the coating’s preferred growth direction is in the (111) direction. Additionally, the coating’s golden color (Figure 5c) and analysis of the XRD results against reference patterns confirm the presence of the TiN phase on the SS 410 substrate. The coating exemplifies TiN’s cubic structure and polycrystalline nature. The analysis results of coatings generated by the CAE method showed the presence of expected phases on the 410 stainless steel substrate.

### 3.2. SEM Observation

**Figure 6:**
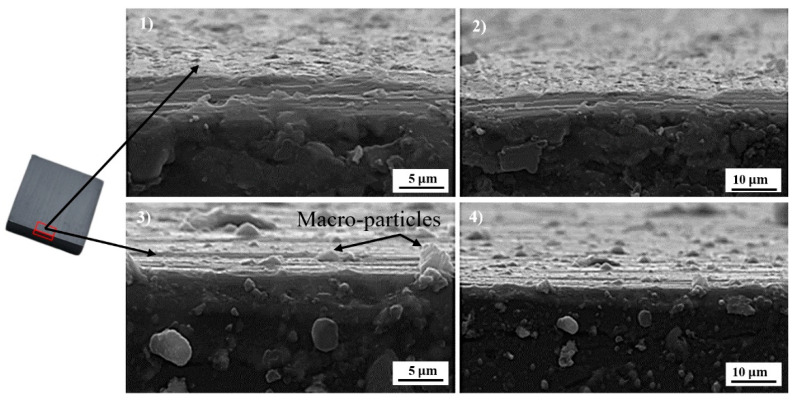
The surface morphology of CrN (No. 1 and 2) and TiN (No. 3 and 4) on steel substrates as determined by CAE at magnifications of 2500× (**right**) and 5000× (**left**).

Figure 6 shows the surface morphology of the CrN and TiN coatings created by the CAE method on the SS 410 substrate in two magnifications of 2500× and 5000×. As seen in Figure 6, there were no pinholes, which confirmed the precise coating process using PVD [41]. The microstructure of CrN coating is more uniform than that of TiN coating, and macro-sized particles are less visible on its surface. Consequently, the average surface roughness parameters were lower than those in the TiN-deposited case [23].

**Figure 7:**
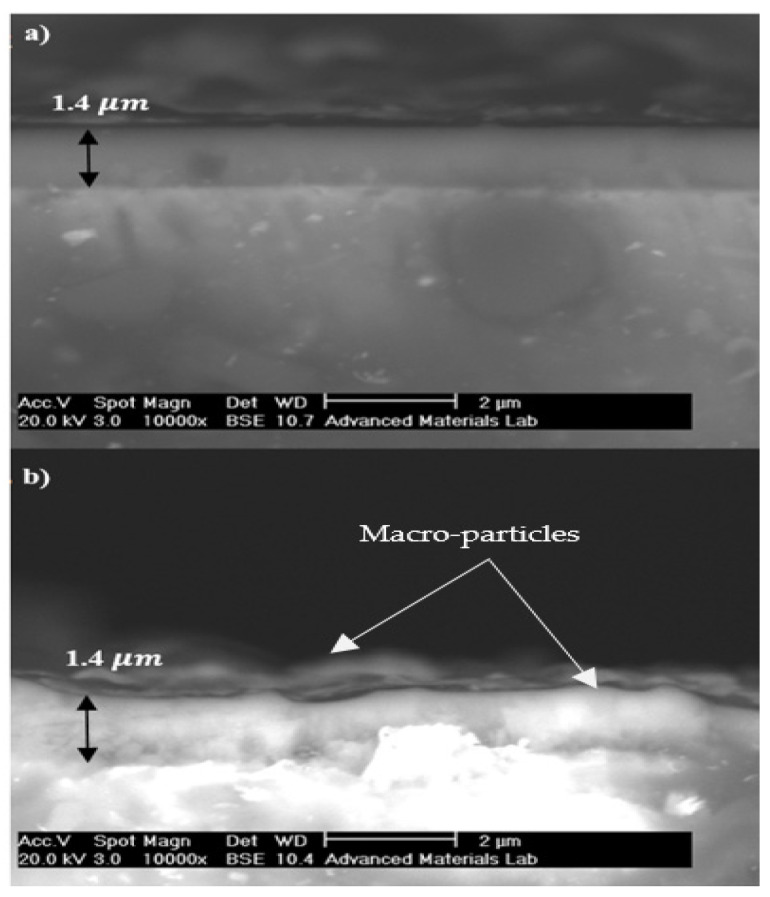
SEM images of the cross-sections of (**a**) CrN-coated layer and (**b**) TiN-coated layer.

Cross-section SEM micrographs (Figure 7) of TiN and CrN layers show the compact structures without delamination and cracking, which confirmed a proper adhesion between the substrate (SS 410) and coatings. The thickness of layers was found to be approximately 1.4 µm. As illustrated in Figure 7a, the CrN-coated layer BPP is smoother than the TiN-coated layer due to the absence of macro-particles. Figure 7b shows the macro-particles cause non-uniformity of the coating surface. In terms of their composition, macro-particles are rich in metals and low in nitrogen. This fault is due to the surrounding coated samples and serves as a suitable location for diffusion of ions present in the electrolyte in contact with CAE coated metals, such as chloride. This leads to local corrosion attacks [41].

### 3.3. EDX Analysis

**Figure 8:**
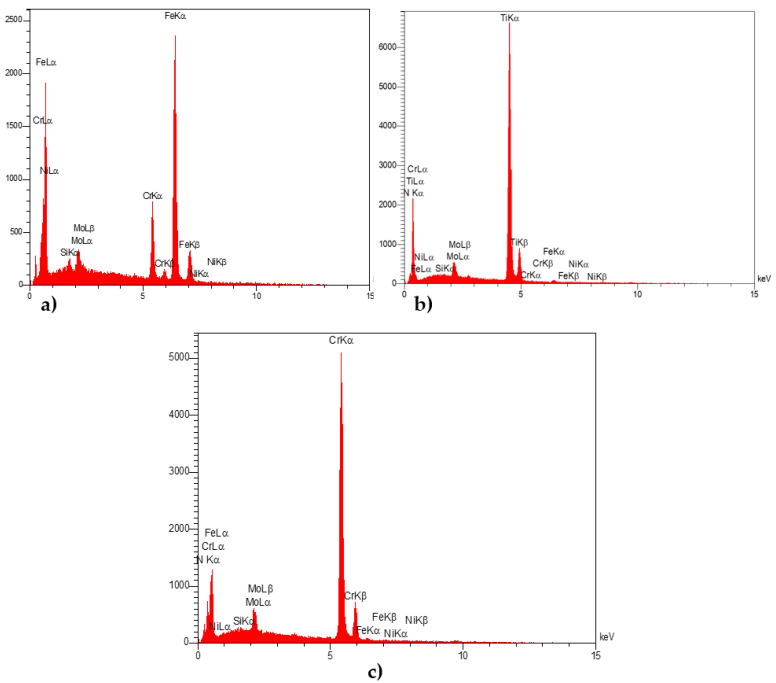
EDX patterns on the surface of different materials, including (**a**) SS 410 as a substrate, (**b**) TiN-coated specimens, and (**c**) CrN-coated specimens.

EDX patterns were obtained to confirm the formation, homogeneity, and distribution percentage of different materials, including SS 410, and TiN and CrN coatings, for various specimens. EDX analysis of TiN and CrN coatings reveals the uniform distribution of elements (the errors are reported in Table 4). Figure 8 demonstrates the EDX patterns of SS 410, TiN, and CrN. The weight percentage of elements based on excitation energy for K LINE [42] as a function of the atomic no. are shown for all samples.

The weight element percentages were found to be 70.84%, 79.44%, and 78.62% for substrate, TiN, and CrN coatings, respectively. The results of the EDX analysis of the coated and uncoated specimens were completely consistent with those of the XRD analysis.

### 3.4. Corrosion Resistance

#### 3.4.1. EIS Measurement

**Figure 9:**
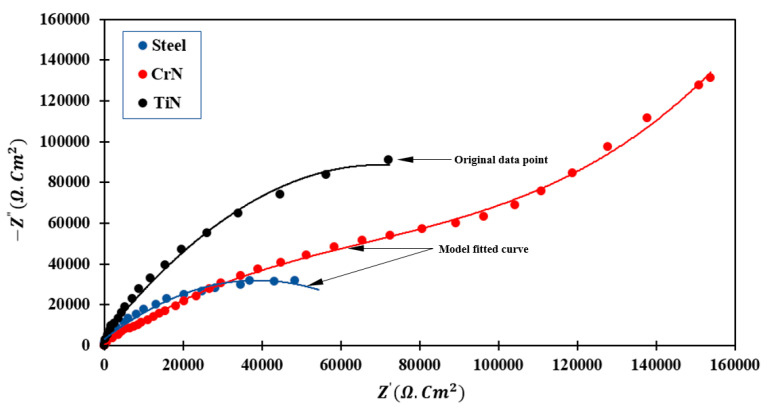
EIS Nyquist plot of different specimens in Ringer’s solution.

Figure 9 shows the EIS diagrams of various specimens after 30 min of immersion in Ringer’s solution. The impedance data were collected using a simple fit model, which resulted in a proper equivalent circuit composed of real and imaginary components [15]. The samples in this study adhered to the Constant Phase Element (CPE) model [16]. In the case of CrN-coated specimens, as defined by the model fitted curve, the formation trend is more extended, resembling semicircles, due to the coating’s surface activity. The formation of layers in contact with the electrolyte solution decreased the electrochemical reaction rate, thereby increasing corrosion resistance [17]. The semicircle is crushed due to the electrode’s heterogeneous surface. At low frequencies, however, a diffusion process results in the formation of the second semicircle. That is, the CrN-coated specimen has two semicircles, whereas the TiN-coated and uncoated specimens each have a single semicircle. This means that the CrN-coated specimen has more barriers to transmitting electrolytes to the substrate, which led to a decrease in the speed of electrochemical reactions and consequently increased resistance of corrosion, and this coating has a higher corrosion resistance than other coatings. Moreover, the TiN-coated specimen exhibits a larger semicircle than uncoated steel (higher impedance). This property indicates that the polarization resistance of the TiN-coated specimen is higher than that of uncoated 410 stainless steel. That is, this indicates a higher corrosion resistance of TiN coating compared to the uncoated steel, due to the formation of a passive surface layer on the coating. The high Rct values for the TiN coating compared to steel demonstrate that the TiN-coated specimen has higher corrosion resistance. High resistance values result in enhanced resistance against the diffusion of corrosive ions and thus improved coating protection performance. Water and corrosive ions cannot penetrate the coating due to its increased resistance and decreased porosity. As a result, the contact between the coating’s open porosities, which are in contact with the electrolyte and are frequently referred to as pin holes, is significantly reduced. As a result, the possibility of corrosive attack decreases, resulting in increased corrosion resistance.

#### 3.4.2. PDP Test

**Figure 10:**
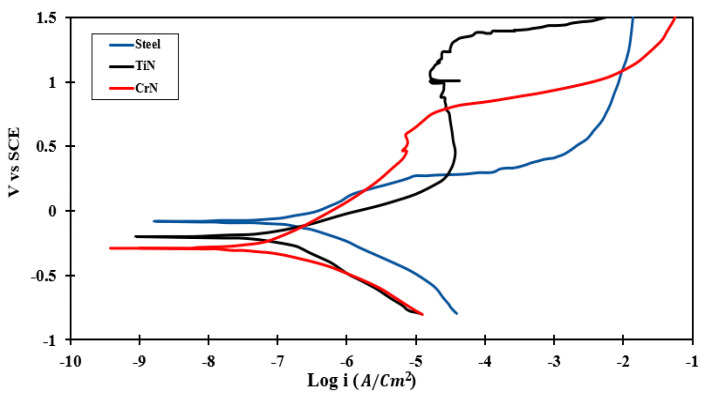
Corrosion behavior of various specimens in the PDP test.

Figure 10 shows the PDP diagrams of various specimens. Table 5 also includes the parameter values obtained from PDP curves in Tafel region analysis for various specimens. To compare the corrosion behavior of samples based on the PDP test, two parameters of $E_{corr}$ and $i_{corr}$, which are the measured corrosion resistance from thermodynamic and kinetic aspects [34], respectively, were introduced. The corrosion potential of SS 410 was found to be −78.3 mV vs. SCE, and its curve was 120–200 vs. SCE. Larger positive values were found compared to those of the uncoated samples. The main reason for this trend of bare SS relative to the coated specimens is due to corrosion product formation, such as iron oxide on top of the surface at the reaction initiation. However, $i_{corr}$ values of TiN- and CrN-coated specimens are slightly lower than that of uncoated SS 410. According to the data in Table 5, the minimum amount of $i_{corr}$ is related to CrN. In addition, the corrosion rate in the CrN-coated specimen is significantly greater than that of TiN-coated and uncoated specimens (using CrN coating on 410 stainless steel as a substrate improves corrosion resistance by up to 3 times). Further analysis of the Tafel sections exhibited that the cathodic branch slope (*β_C_*) varies from 203 to 300 mV/dec. The reduction in oxygen in coated specimens shows the dominance of the cathodic process. The anodic branch slope (*β_a_*) varies from 164 to 339 mV/dec. Comparison of $\beta_{a}$ and $\beta_{C}$ proved that the corrosion procedures were under cathodic control [43,44]. Polarization resistance is given in Equations (4) and (5), which include the terms of $i_{corr}$ and $E_{corr}$_._ Accordingly, the corrosion resistance can be analyzed kinetically and thermodynamically. The results of the corrosion resistance test indicated that the CrN-coated specimen has the highest corrosion resistance, and the lowest corrosion rate is due to its highest polarization resistance ($R_{p}\;$= 690.6 Ω·cm^2^). The $R_{p}$ value was calculated using Equation (5) and is equal to 410.2 Ω.cm^2^ for the TiN-coated sample.

(4) $$\beta =\frac{\beta c\;\cdot \;\;\beta a}{2.3\;\left(\beta c+\beta a\right)}$$

(5) $$R_{P}=\frac{\beta }{\mathrm{icorr}\;}$$

The number of surface pores is a very important feature of materials, and affects corrosion behavior in coated samples [45,46]. The pore parameter can be evaluated using Equation (6). In this equation, F is the number of the pores, $R_{PS}$ is the bare SS polarization, $R_{P}$ is coated sample’s polarization resistance, and ΔE is the difference in the corrosion potential of bare SS and coated samples. $\beta_{a}$ is the slope of the bare SS anodic branch.

(6) $$F=\frac{R_{PS}}{R_{P}}10^{\frac{\Delta E}{\beta_{a}}}$$

The obtained results showed that the lowest pore density is related to CrN-coated samples with the value of 2.72%. This value is 4.58% for TiN-coated specimens. Therefore, CrN is more dense and has greater structural integrity.

In the TiN-coated specimen, the corrosion potential and corrosion current were increased compared to those of the uncoated sample. The anode in these specimens exhibits a greater range of passive states than the uncoated substrate. As the potential increases, the coating exhibits a greater proclivity to become passive. This is due to the formation of a passive Cr_2_O_3_ layer in the Cr base coating, which prevents corrosion at the layer’s interface and coating.

Moreover, compared to previous studies, the corrosion current of CrN-coated steel using the PVD method with CAE technology is significantly less than the corrosion current of CrN-coated steel using the PF [15] and PVD [16] methods. Moreover, the corrosion current of TiN-coated steel produced by the PVD method employing CAE technology is significantly lower than the corrosion current of TiN-coated steel produced by the EBPVD [17] method.

**Figure 11:**
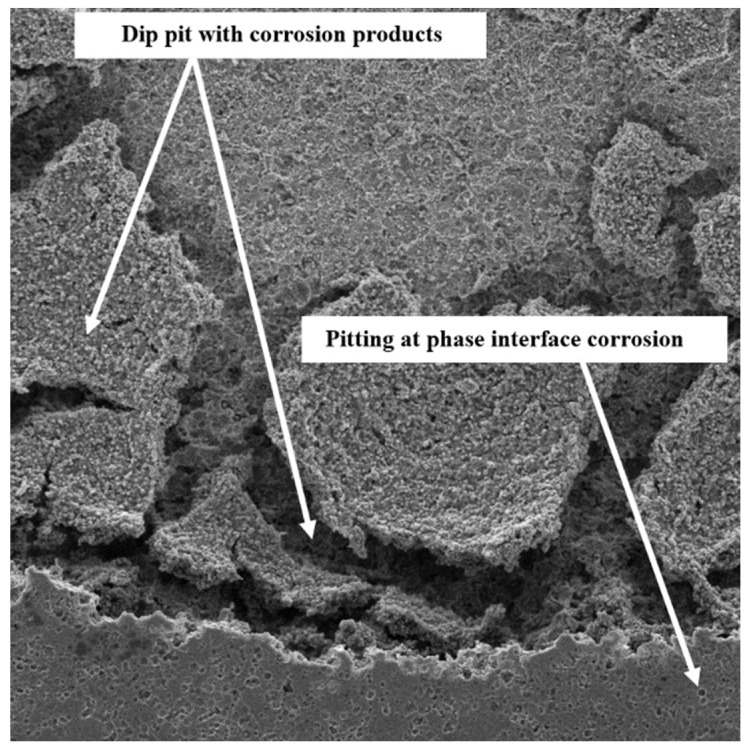
SEM image (1000×) of the uncoated specimen’s surface following the corrosion test.

**Figure 12:**
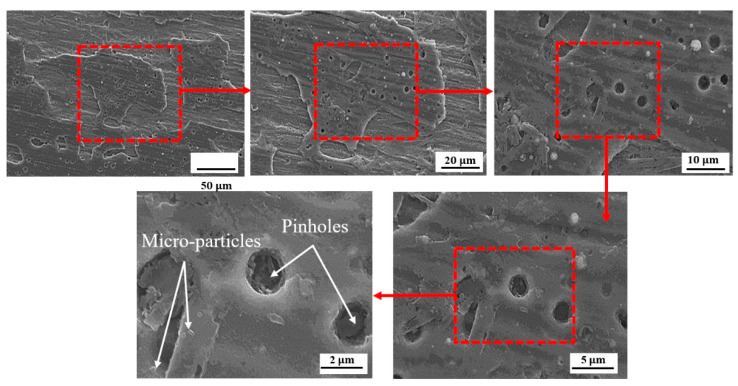
Surface analysis of the CrN-coated specimen following the CAE corrosion test.

**Figure 13:**
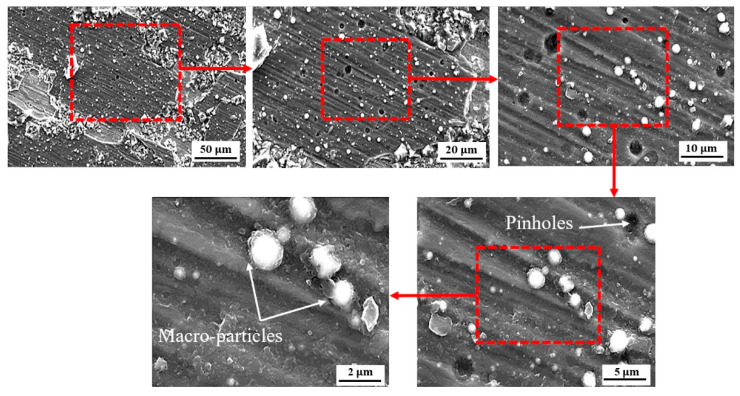
Surface analysis of the TiN-coated specimen following the CAE corrosion test.

Figure 11, Figure 12 and Figure 13 illustrate the surface morphology of the uncoated, TiN-coated, and CrN-coated specimens, respectively. The bare SS had the highest corrosion on the surface, resulting in damage to local passive films. This is referred to as pitting corrosion. Fragments of metal surfaces dissolved instantly as a result of this phenomenon. The uncoated stainless steel surface is covered in corrosion products and deep pits. Moreover, several small pits at the phase interface were determined to be more corrosive to SS 410 than other samples in Ringer’s solution. Corrosion products are visible alongside droplet-like structures in the TiN-coated specimen. There is less defect dispersion and corrosive production on the surface of the CrN-coated specimen in Figure 12. Corrosion tests and SEM images corroborated these assertions. Figure 13 shows the details of the corrosion products produced by TiN-coated samples. This is demonstrated by the presence of more corrosion products and droplets than with CrN-coated surfaces.

As shown in Figure 12 and Figure 13, defects such as pin holes and macro-particles are the main factors that can affect the resistance corrosion of the coated samples at the lower anodic potential. The lower dispersion of the CrN surface is shown in Figure 12. Figure 13 illustrates more localized corrosion caused by macro-particles in TiN-coated specimens. Accordingly, there is no cracking on the surface of the CrN-coated sample. Surface SEM images proved that the sample coated with CrN has fine smooth crystallites, a dense structure, and some micro-particles without pores and cracks.

### 3.5. ICR

**Figure 14:**
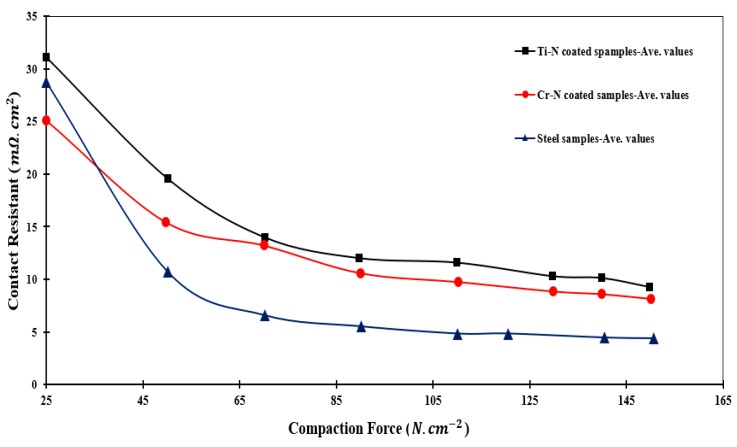
The comparison of the ICR of various specimens including uncoated SS, TiN-coated 410 SS, and CrN-coated 410 SS [23].

Changes in ICR against pressure for various specimens (uncoated SS, CrN-coated SS, and TiN-coated SS) are shown in Figure 14. It is evident that ICR decreases as applied pressure increases in all cases. This is due to the increased effective contact surface. Furthermore, the ICR of coated specimens is greater than that of the uncoated sample. For this purpose, the ICR values of the uncoated SS, CrN-coated SS, and TiN-coated SS are 8.34, 9.26, and 11.23 mΩ·cm^2^, respectively. CrN samples have a lower ICR than TiN samples.

Corrosion rate $({µA\;\mathrm{cm}}^{-1}>1)\;$ and electrical contact resistance $\left(mΩ\cdot {\mathrm{cm}}^{2}>10\right)$ are considered by the US Department of Energy to be the industry standard characteristics for bipolar plates [47]. In light of this, the value of CrN-coated 410 SS is acceptable for use in BPPs. Contact resistance measurements on both TiN and CrN coatings revealed values closer to those defined by the US Department of Energy. The current study’s ICR of the CrN-coated specimen is nearly half that of the CrN-coated steel obtained via PF [32] and PVD [32,33] methods, equal to 20 mΩ·cm^2^. Additionally, the ICR of TiN-coated steel in this study is approximately one-third that of the ICR of TiN-coated steel obtained through EBPVD [48].

## 4. Conclusions

The 410 SS alloy was selected for the bipolar plates in this study, and was coated with an equal thickness of TiN and CrN using the CAE method. EIS and PDP methods were used to conduct electrochemical tests in Ringer’s solution. Corrosion resistance and ICR were determined for each case to determine their suitability for use in a fuel cell environment. As shown in Table 1 and based on the latest developments, the study’s findings are as follows:

- The corrosion resistance of TiN-coated and CrN-coated specimens is significantly greater than that of uncoated steel, thus reducing contamination, extending the life of the bipolar plates in fuel cells, and lowering their maintenance costs.
- According to PDP results, CrN coated on 410 SS exhibited a maximum value of $R_{p}$ (690.6 $\Omega \cdot {\mathrm{cm}}^{2}$) and a minimum rate of corrosion (0.032).
- The results of the EIS demonstrate that the TiN-coated specimen has a larger semicircle than the uncoated steel specimen (higher impedance). This property indicates that the TiN-coated sample has a higher polarization resistance than that of uncoated 410 stainless steel.
- In CrN-coated samples, the formation of more continuous semicircles of larger diameter is due to surface activity and the formation of layers in contact with the electrolyte solution, which slows the electrochemical reaction. The deepening of the semicircle is related to the non-uniformity of the electrode surface.
- The SEM images confirmed that CrN crystallites are smooth and without cracks. This increases its resistance to corrosion in the fuel cell environment compared to other TiN and bare SS.
- The average values of ICR for CrN and TiN samples were found to be 9.26 and 11.23 mΩ·cm^2^, respectively. This substantiated the superior performance of CrN-coated layers for PEM fuel cell BPPs.
- Due to its ICR, corrosion resistance, low material cost, and ease of manufacture, the CrN-coated 410 SS may be an excellent candidate for use in PEM fuel cells as a BPP.

## Figures and Tables

**Table 1:** Characteristics of CrN and TiN coatings used for BPPs in fuel cells.

Ref No.	Substrate as Bipolar Plates	Coated Samples (TiN and CrN)	Other Coating Study	Surface Morphology	XRD and EDS	Coating Methods	Corrosion Behavior	ICR
[30]	316L SS	✓✕	TiCN	✓	✓✓	Closed Field Unbalanced Magnetron Sputter	✓	✓
[31]	Titanium	✓✕	✕	✓	✓✕	Multi-arc ion plating	✓	✓
[32]	316L SS	✕✕	TiCN	✓	✓✓	Mathode glow discharge plasma	✓	✕
[23]	410 SS	✓✓	✕	✕	✕✕	CAE	✕	✓
[30]	316L SS	✓✕	TiCN	✓	✓✓	Closed Field Unbalanced Magnetron Sputter	✓	✓
[33]	410 SS	✕✕	TiN/TiAlN	✓	✓✓	Arc ion plating system	✓	✕
[34]	Al 7000	✓✓	✕	✓	✓	Magnetron sputtering	✓	✓
This study	410 SS	✓✓	✕	✓	✓	CAE	✓	✓

**Table 2:** Chemical composition of 410 stainless steel sheets (wt.%) [23].

C	Mn	Si	P	S	Cr	Ni
0.13	0.95	1	0.03	0.03	13	0.70

**Table 3:** Ringer’s solution chemical composition.

Content	Concentration (g/100 mL)	mEq/L
Sodium chloride	0.85	-
Potassium chloride	0.03	-
Calcium chloride anhydrous	0.33	-
Sodium	-	147
Potassium	-	4
Calcium	-	4.5
Chloride	-	156

**Table 4:** EDX data from the coated and uncoated specimen surfaces.

Specimen	Elt.	Int.	K	Kr	W%	A%
Uncoated samples	C	24.0	0.0212	0.0195	7.23	26.30
	Si	15.5	0.0150	0.0138	2.00	3.11
	Cr	159.0	0.1884	0.1733	15.63	13.14
	Fe	594.3	0.7386	0.6796	70.84	55.44
	Ni	1.2	0.0016	0.0015	0.16	0.12
	Mo	35.1	0.0352	0.0324	4.14	1.89
TiN-coated samples	N	192.9	0.0911	0.0809	14.11	35.07
	Ti	1424.0	0.8544	0.7590	79.44	57.76
	Fe	9.4	0.0062	0.0055	0.62	0.39
CrN-coated samples	N	76.0	0.0420	0.0364	8.91	24.16
	Cr	1186.0	0.8711	0.7560	78.62	57.40
	Fe	8.4	0.0065	0.0056	0.63	0.43

**Table 5:** The parameters determined by Talfel analysis for various specimens.

Sample	*E_corr_* (mV vs. SCE)	*i_corr_* (µA/cm^2^)	*β_C_* (mV/dec)	*β_a_* (mV/dec)	Corrosion Rate (mpy)	R_p_ (Ω·cm^2^)
Base steel	−78.3	0.18	220	220	0.096	230.1
CrN	−279.5	0.07	203	339	0.032	690.6
TiN	−192.9	0.11	300	164	0.060	410.2

## Data Availability

The data that support the findings of this study are available from the corresponding author upon reasonable request.
